# 2 Hz EA Reduces Heroin Withdrawal-Induced Hyperalgesia and Heroin Relapse by Downregulating P2X3 Receptors in DRG Neurons

**DOI:** 10.1155/2019/1873859

**Published:** 2019-12-24

**Authors:** Lin Chen, Changlong Leng, Xiaokang Gong, Baomiao Ma, Qin Ru, Qi Xiong, Mei Zhou, Xiang Tian, Kai Yue, Chaoying Li, Yuxiang Wu

**Affiliations:** ^1^School of Physical Education, Jianghan University, Wuhan 430056, China; ^2^Wuhan Institutes of Biomedical Sciences, Jianghan University, Wuhan 430056, China

## Abstract

Electroacupuncture (EA) has effective analgesic effects. Our previous study demonstrated that the upregulation of P2X3 receptors in the dorsal root ganglia (DRG) might participate in heroin withdrawal-induced hyperalgesia. The aim of this study is to further explore whether 2 Hz EA reduces heroin relapse associated with its analgesic effect and whether P2X3 receptors in the DRG are involved in this process. 2 Hz EA was adopted to treat the heroin SA rats in the present study. Heroin-seeking and pain sensitivity were evaluated. The expression of P2X3 receptors in the DRG was detected. Our results showed that compared with the control group, the reinstatement, thermal hyperalgesia, and mechanical allodynia of the heroin-addicted group were increased significantly. The expression of P2X3 receptors in the DRG was increased markedly. After being treated using 2 Hz EA, reinstatement was reduced, hyperalgesia was decreased, and the upregulated expression of P2X3 receptors in the DRG had decreased significantly compared to that in the heroin-addicted group. Consequently, our results indicated that 2 Hz EA was an effective method for treating heroin-induced hyperalgesia and helping prevent relapse, and the potential mechanism might be related to the downregulation of P2X3 receptor expression in the DRG.

## 1. Introduction

The essential problem in the treatment of opioid (including heroin) addiction is the high rate of relapse [1]. The neurobiological mechanisms underlying opioid relapse and cravings are complex [2]. Opioid addiction is generally considered the biological basis of “psychological” dependence, while withdrawal symptoms, especially abnormal pain, contribute to drug cravings [3, 4]. Clinical investigations have also demonstrated that hyperalgesia after long-term opiate exposure might induce drug cravings during prolonged opiate abstinence [5]. Results have indicated that hyperalgesia induced by opioid withdrawal might be one of the important causes of opioid relapse and craving.

Several periphery molecular targets have been proposed to understand the hyperalgesia induced by opioid withdrawal, such as morphine receptors (MORs) [6, 7], N-methyl-D-aspartate receptors (NMDA-R) [8], and transient receptor potential vanilloid-1 (TRPV1) [9]. However, the precise mechanism underlying opioid withdrawal-induced hyperalgesia is still unclear. Type 2X purinergic receptors (P2X) are part of the P2 purinergic family of proteins and the ligand-gated cation channels [10]. All P2X subtypes are found on sensory neurons, and P2X3 receptors have the highest level of expression (in terms of both mRNA and protein) on small nociceptive sensory neurons in dorsal root ganglia (DRG) [11]. Observations with antagonists selective for P2X3 receptors [12] and models of P2X3 receptor knockout mice [13] have confirmed that P2X3 receptors play an important role in chronic neuropathic pain [14]. Many studies have found that P2X3 receptors are upregulated in the DRG of chronic neuropathic pain models [15]. In addition, we demonstrated that the expression of P2X3 receptors in the DRG was increased obviously in the development of peripheric hyperalgesia induced by heroin withdrawal in heroin SA rats [16].

Electroacupuncture (EA) is a procedure in which fine needles are inserted into an individual at discrete points, and then electrical stimulation is applied, aiming to relieve chronic pain [17]. Our previous study and other reports have demonstrated that 2 Hz EA has analgesic effects *in vivo* [18, 19]. The USA National Acupuncture Detoxification Association reported that EA could prevent the development of morphine tolerance in rats [20]. Previous studies by our groups and others also found that 2 Hz EA treatment could significantly reduce conditional cue-induced heroin-seeking in heroin SA rats [21, 22].

There is still a lack of evidence to support the notion that 2 Hz EA reduces heroin relapse via its analgesic effect by affecting the expression of P2X3 receptors in the DRG. Therefore, in the present study, 2 Hz EA was adopted to treat heroin SA model rats. The rate of heroin relapse was evaluated with a reinstatement experiment, and the pain sensitivities were examined using paw thermal and mechanical withdrawal tests. The expression of P2X3 receptors in the DRG was observed using immunofluorescence. Our results will partly elucidate the analgesic effect of 2 Hz EA that may assist in suppressing heroin relapse and the potential mechanism through P2X3 receptors in the DRG.

## 2. Materials and Methods

### 2.1. Animals

Male Sprague Dawley rats, weighting 275–300 g at the beginning of the experiments, were purchased from Vital River Laboratory Animal Technology Co., Ltd, Beijing, China. The animals were maintained under a 12 h reversed light/dark cycle (with darkness starting from 8:00 am) with controlled room temperature and humidity. Tap water was made available *ad libitum*, and food was restricted to 20 g per day to keep animal weight constant [23, 24]. All procedures were performed in accordance with the National Institutes of Health Guide for the Care and Use of Laboratory Animals and with the approval of Animal Care and Use Committee of Jianghan University.

### 2.2. Heroin Self-Administration Rat Model

The heroin self-administration rat model that represented heroin relapse behaviours and was used to induce hyperalgesia was developed with a progressive fixed ratio programme. Experimental apparatus, procedures for surgery, and self-administration training were described in our previous report [23].

In short, after a vein intubation operation, heroin SA training was carried out in operant chambers encased in sound- and light-attenuating cubicles, which were equipped with fans that provided ventilation (AniLab Software & Instruments Co., Ltd., Ningbo, China). Animals were allowed to self-administer heroin (30 *μ*g/kg per infuse) under a fixed ratio 1 (FR 1) schedule of reinforcement, and we observed nose pokes for 3 h each day. The acquisition sessions were carried out until stable heroin intake was reached (typically within 10–12 days). Twenty-four rats developed a stable pattern of heroin intake. Thereafter, these rats were randomly divided into three groups (*n* = 8 per group): a heroin-addicted group, a sham EA group, and a 2 Hz EA group. The same experimental procedures were used for the rats in the control group (*n* = 8) except the heroin was substituted with the same volume of saline.

### 2.3. Extinction and 2 Hz EA Treatment

The rats were abstinent from heroin for two weeks, during which time they lived in their individual home cages. Heroin-reinforced behaviours can be extinguished, which, in most cases, took place around days 12–14 after abstinence from heroin. At the same time, the rats in the 2 Hz EA group were treated with a 2 Hz EA stimulus (1 mA and 0.1 ms pulse width) for 30 min on the zusanli (ST36) and sanyinjiao (SP6) acupoints every day during the extinction period according to methods reported in the literature [25]. In other words, rats were kept in special canvas holders with their hind legs and tails exposed. Two 0.3 mm diameter stainless steel needles were inserted into each hind leg in the acupoints ST36 (5 mm lateral to the anterior tubercle of the tibia) and SP6 (3 mm proximal to the superior border of the medial malleolus, at the posterior border of the tibia). The anatomical location of acupuncture points stimulated in rats corresponded to the acupoints in rat as described previously [21]. Constant current electric stimulation produced by modified current-constant Han's Acupoint Nerve Stimulator (LH202; Huawei Co Ltd, Beijing, China) was administered via the two needles. The frequency of stimulation used was 2 Hz. The intensity of the stimulation was 1.0 mA, lasting for 30 min. Rats of the sham EA group were treated with an acupuncture stimulus for 30 min on the same acupoints every day during the extinction period without electrical stimulation.

### 2.4. Reinstatement

After extinction, cue-induced reinstatement was examined to verify whether the addictive behaviour was successfully induced. During testing, the rats were reintroduced to the operant cages for 2 h training. At the beginning of the training, the rats were administered a conditioned cue priming (the house light extinguished, and the injection pump noise and the red nose poke light were turned on). A single active nose poke was obtained with conditioned cue priming, but no heroin injection. The number of active nose pokes and inactive nose pokes was recorded.

### 2.5. Nociceptive Behavioural Test

Thermal and mechanical nociceptive responses were used to verify whether nociceptive behaviour was induced and assessed using a dynamic plantar instrument with a radiant heat source and von Frey hairs, respectively (UGO Basile, Comerio, Italy). Evaluation of paw withdrawal thresholds (PWTs) was performed according to the methods described in the literature [19]. Concretely, the rats were acclimatized to the apparatus that consisted of three individual perspex boxes on a glass table. A mobile radiant heat source was located under the table and focused onto the desired paw, and the heat was increased gradually until a withdrawal response was evoked, and the latency of heat needed to cause the withdrawal response was recorded. In order to prevent tissue damage, an automatic cutoff at 30 sec was set. Rats were also placed on a wire mesh floor in clear cylindrical plastic enclosures. Following 20 min of acclimation, a von Frey filament was placed on the plantar surface of the right hind paw and the force was increased gradually until a withdrawal response was evoked, and the amount of force needed to cause the withdrawal response was recorded. The threshold, expressed in second or grams, was considered the time or force that induced a withdrawal response, with a 30 s or 50 g limit. Each trial was repeated 3 times at approximately 5-minute intervals, and the mean value to produce a withdrawal response was determined.

**Figure 1:**
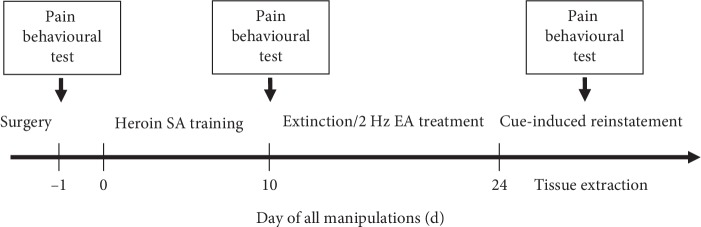
Experimental timeline and diagram of the heroin self-administration (SA) and pain behavioural tests (thermal and mechanical nociceptive responses). Pain behavioural tests were performed on d −1 prior to heroin self-administration, on d 10 prior to extinction and 2 Hz EA treatment, and after d 24 cue-induced reinstatements.

Testing was conducted before drug exposure (baseline, BL), extinction/EA treatment, and after cue-induced reinstatement. The experimental timeline and details are illustrated in Figure 1.

### 2.6. Immunohistochemical Staining

Immediately after nociceptive behavioural testing, the rats were systemically perfused with 4% paraformaldehyde during 2–3% isoflurane-induced anesthesia. The L5-6 DRG were then isolated and postfixed overnight in 4% paraformaldehyde in PBS. After paraffin embedding, DRG paraffin tissue blocks were cut into 4 *μ*m thick slices. After dewaxing and rehydration, microwave antigen retrieval, and endogenous peroxidase blocking, slices were incubated with rabbit anti-P2X3 (1 : 200 dilution; Alomone Labs, Jerusalem, Israel) and diluted in PBS overnight at 4°C. After 3 rinses in PBS, the sections were incubated with fluorescent secondary antibody (1 : 200 dilutions; Abcam, Cambridge, UK) in the dark at 37°C for 40 min. After three washes again in PBS, sections were mounted and covered with glycerol. The sections were examined with fluorescence microscopy (Olympus BX51, Japan), and photographs were taken with a filter set for Cy3 (excitation 540–580 nm/emission 560–620 nm). The optical densities of P2X3 were counted in a blind fashion on 16–20 randomly selected L5-6 DRG sections from 8 animals per condition. The results are expressed as relative optical density from these sections. Negative controls in which PBS was used instead of the primary antibody were processed in the same manner.

### 2.7. Statistical Analyses

All the results are expressed as the mean ± SEM. The statistical significance of the results was analysed using one-way or two-factor repeated ANOVA. All statistical analyses were performed using SPSS for Windows, version 11.5 (SPSS Inc., Chicago, IL, USA). When significance was found using ANOVA procedures, post hoc analyses were conducted using the Fisher LSD test. *p* < 0.05 was considered statistically significant.

## 3. Results

### 3.1. Effects of 2 Hz EA on Conditional Cue-Induced Heroin Reinstatement

**Figure 2:**
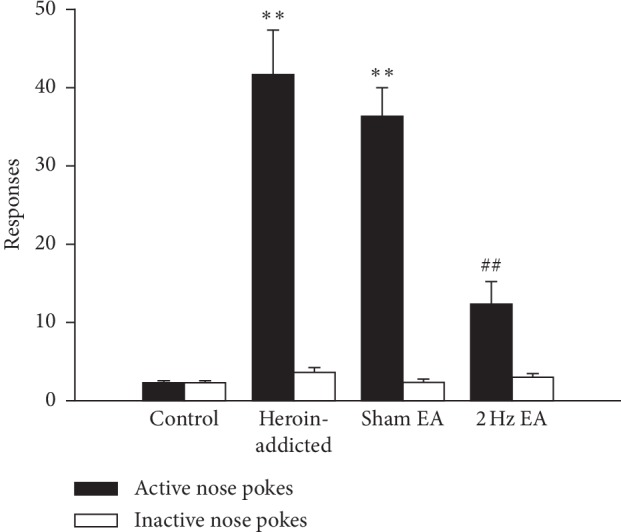
2 Hz electroacupuncture treatment attenuated conditioned cue-induced reinstatement in rats. Values are given in mean ± SEM of eight animals. ^*∗∗*^*p* < 0.01, as compared with the control group; ^##^*p* < 0.01, as compared with the heroin-addicted group.

The heroin self-administration rat model represented heroin relapse behaviours, which were developed with a progressive fixed ratio programme. The experimental apparatus, procedures for surgery, and self-administration training followed our previous report [23]. As shown in Figure 2, the number of active nose pokes (representing conditional cue-induced reinstatement) significantly increased in the heroin-addicted group and sham EA group when compared to that in the control group (*p* < 0.01). The number of active nose pokes significantly decreased in the 2 Hz EA group compared with that in the heroin-addicted group (*p* < 0.01). In contrast, no significant differences (*p* > 0.05) were observed in the number of inactive nose pokes during reinstatement among the control, heroin-addicted, sham EA group, and 2 Hz EA groups, which suggested that rats retained good discrimination between active nose pokes and inactive nose pokes. These results indicated that a successful heroin-addicted rat model was verified, and 2 Hz EA, but not sham EA treatment, significantly reduced the conditional cue-induced reinstatement behaviours in heroin-addicted rats.

### 3.2. Effects of 2 Hz EA on Thermal and Mechanical Hypersensitivity

**Figure 3:**
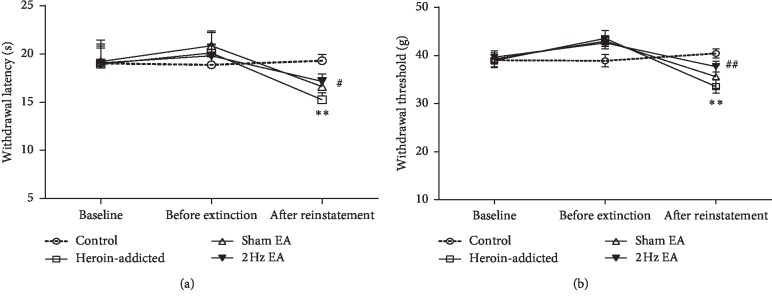
2 Hz electroacupuncture treatment reversed the thermal (a) and mechanical (b) hypersensitivity after reinstatement in rats. Values are given in mean ± SEM of eight animals. ^*∗∗*^*p* < 0.01, as compared with the control group; ^#^*p* < 0.05; ^##^*p* < 0.01, as compared with the heroin-addicted group.

Hyperalgesic responses were measured on the d −1 prior to heroin self-administration (baseline), on d 10 prior to extinction and 2 Hz EA treatment, and after d 24 cue-induced reinstatements. As shown in Figure 3, whether it is on d −1 or on d 10, there were no significant group differences so as to the thermal nociceptive threshold and mechanical withdrawal threshold.

As illustrated in Figure 3(a), after reinstatements, the thermal nociceptive threshold of heroin-addicted rats was significantly decreased compared with that of the control group (*p* < 0.01), indicating the development of thermal hypersensitivity. Interestingly, the thermal threshold in the 2 Hz EA-treated group increased significantly compared with that in the heroin-addicted rats (*p* < 0.05). Similar results were observed in mechanical withdrawal threshold measurements. As illustrated in Figure 3(b), the mechanical withdrawal threshold of heroin-addicted rats was significantly decreased compared with that of the control group (*p* < 0.01), indicating the development of mechanical hypersensitivity. Similarly, the mechanical thresholds in the 2 Hz EA-treated group also increased significantly compared with those in the heroin-addicted rats (*p* < 0.01). There was no significant difference in either thermal or mechanical withdrawal threshold between the sham EA group and the heroin-addicted group (*p* > 0.05). Our results indicated that 2 Hz EA treatment could significantly reduce hyperalgesic behaviours in heroin-addicted rats.

### 3.3. Effects of 2 Hz EA on Expression of P2X3 Receptors

**Figure 4:**
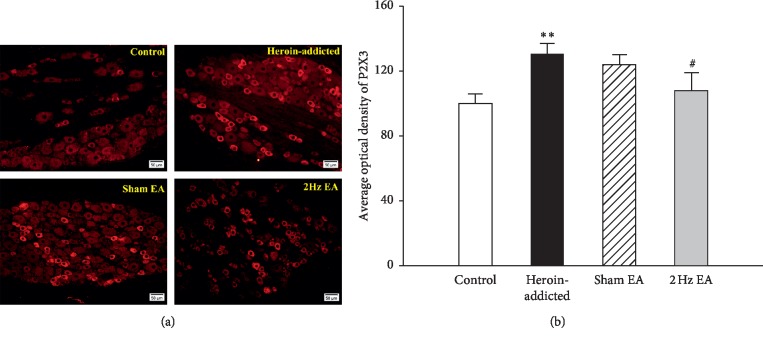
The differences of P2X3 receptor expressions in L5∼6 DRGs neurons in the different groups. The immunofluorescence pictures (a) and histogram (b) of P2X3 receptors showed that expressions of P2X3 receptors increased in heroin-addicted rats and reduced in the EA group. Values are given in mean ± SEM of eight animals. ^*∗∗*^*p* < 0.01, as compared with the control group; ^#^*p* < 0.05, as compared with the heroin-addicted group. The scale is 50 *μ*m.

P2X3 receptor expression in the L5∼6 DRG neurons was observed using immunofluorescence (Figure 4). Immunofluorescence pictures (Figure 4(a)) and image analysis (Figure 4(b)) showed that the expression of the P2X3 receptor (average optical density) increased in heroin-addicted rats compared with that in the control group (*p* < 0.01) (*n* = 8 for each group). The average optical density of the P2X3 receptor in the 2 Hz EA therapy group reduced significantly compared with that in the heroin-addicted rats (*p* < 0.05). The P2X3 downregulation effect in the sham EA group was not obvious (*p* > 0.05). It was suggested that 2 Hz EA treatment could significantly reduce the upregulated expression of P2X3 receptors in the DRG in heroin-addicted rats.

## 4. Discussion

There is still a lack of evidence to support the notion that 2 Hz EA reduces relapse in heroin SA rats and that this relapse reduction is associated with the analgesic effect that occurs via affecting the expression of P2X3 receptors in the DRG. In the present study, 2 Hz EA was used to treat heroin SA model rats. We demonstrated that 2 Hz EA, but not sham EA, reduced the hyperalgesia induced by heroin and contributed to prevention relapse. This effect was partially associated with the downregulation of the expression of P2X3 receptors in the DRG. The neurobiological mechanisms underlying opioid relapse and craving are complex [2]. Generally, both the mesocorticolimbic dopamine system and the nigrostriatal dopamine system contribute to drug-seeking [26]. Accumulated data have demonstrated that hyperalgesia induced by opioid withdrawal might be one of the important reasons for opioid relapse and craving [3–5, 27, 28]. Our previous study used a heroin SA rat model, which is similar to the addictive behaviours of drug addicts [29], to provide evidence of hyperalgesia in heroin relapse rats [16]. EA is applied to relieve several kinds of chronic pain in traditional Chinese medicine. It was demonstrated that EA had an analgesic effect on different types of pain *in vivo* [18, 19]. In the animal model, 2 Hz EA significantly attenuates morphine-induced conditioned place preference (CPP) [30] and behavioural sensitization [31]. Yang et al. [32] and Yoon et al. [33] reported that 2 Hz EA can suppress morphine and ethanol self-administration. Studies by our group and others have also found that 2 Hz EA treatment could significantly reduce conditional cue-induced heroin-seeking in heroin SA rats [21, 22]. Therefore, although other electroacupuncture frequencies (such as 100 Hz) may also have a certain analgesic and prevention effect on relapse [34], only the influence of low frequency (2 Hz) has been investigated in our present study.

In the present study, the effects of 2 Hz EA on reinstatement and thermal and mechanical hypersensitivity were chosen to explore whether 2 Hz EA could inhibit heroin-induced hyperalgesia and suppress cravings.

In fact, we found that the thermal and mechanical hyperalgesia in the 2 Hz EA-treated group were decreased significantly compared with that in heroin-addicted rats. Additionally, 2 Hz EA treatment significantly reduced conditional cue-induced reinstatement behaviour in heroin-addicted rats. These data suggested that 2 Hz EA could inhibit hyperalgesia induced by heroin withdrawal and reduce relapse behaviours in heroin-addicted rats. P2X3 receptors in the DRG play an important role in nociceptive transduction during chronic neuropathic and inflammatory pain but are seldom discussed in hyperalgesia induced by heroin addiction. Previous work has shown that the process of P2X3 receptor antagonism inhibits inflammatory hyperalgesia and involves the spinal opioid system [35]. In addition, P2X3 receptor antagonists can block and reverse spinal morphine tolerance [36]. It is suggested that the P2X3 receptor might play an important role in opioid- (including heroin-) induced periphery hyperalgesia. Our previous study demonstrated that the hyperalgesia developing in heroin relapse rats and the expression of P2X3 receptors in DRG increased correspondingly [16]. Generally, the inhibitory effects of 2 Hz EA on the expression of the opioid-induced relapse might be mediated by *µ*- and *δ*-opioid receptors, possibly via accelerating the release of enkephalin [30]. Reports also showed that the analgesic effect of EA was closely correlated with purines and purinergic receptors [37]. Furthermore, 2 Hz EA was shown to induce an apparent analgesic effect by inhibiting the expression of P2X3 receptors in DRG neurons of a CCI rat model [38]. The present study found that 2 Hz EA could significantly reduce the expression of P2X3 receptors in the DRG of heroin-addicted hyperalgesic rats. Our results indicated that 2 Hz EA might be an effective method to treat hyperalgesia induced by heroin, and this effect might be closely associated with the inhibition of the expression of P2X3 receptors in the DRG.

It is worth noting that the total DRG cell was further divided into three subgroups: small cell, medium cell, and large cell (the diameter of these cell subgroups were about 10–20 *μ*m, 20–40 *μ*m, and more than 40 *μ*m, respectively). The current type of recorded slow, intermediate (or mixed), and fast types of ATP-activated current was correlated well with cell size and performed different functions and P2X3 mainly expressed on small nociceptive neurons mediated fast types of ATP-activated current according to others [39–41] and our [42] previous research results. In addition, not only neuronal pathways, but Schwann cells, satellite cells in the dorsal root ganglia, even of the peripheral immune system, microglia, and astrocytes also involved in the development of neuropathic pain [43]. Both the expression difference of P2X3 receptor from the perspective of DRG size or from the perspective of satellite glial cells and other nonneurons are all very interesting questions; we will pay further attention to in the follow-up studies.

## 5. Conclusions

In summary, we showed that heroin SA rats exhibited hyperalgesia and had increased the expression of P2X3 receptors in the DRG. 2 Hz EA could effectively inhibit hyperalgesia induced by heroin withdrawal and reduce relapse behaviours in heroin-addicted rats. The expression of P2X3 receptors in the DRG decreased correspondingly. It was possible that P2X3 receptors were potential peripheral targets related to hyperalgesic states induced by heroin withdrawal. 2 Hz EA might be an effective method for treating hyperalgesia induced by heroin withdrawal and suppressing heroin relapse partly via regulating P2X3 receptor expression in the DRG. Further study is needed to elucidate the potential mechanism of 2 Hz EA's effect on relapse.

## Data Availability

Those datasets generated during and/or analysed during the current study are available from the corresponding author on reasonable request.
